# Managing Vulvovaginal Atrophy, in Postmenopausal Women, Without Hormones: Efficacy of a Hyaluronic Acid Vaginal Moisturizer

**DOI:** 10.3390/healthcare14162589

**Published:** 2026-08-18

**Authors:** Maria Alice Pimentel Falcão, Carolina Parga Martins Pereira, Tázia Lopes de Castro, Julia do Valle Brunetti, Alexandre Silva Cunha, Andrea Mendes Taira, Arthur Melo Kummer

**Affiliations:** Medical Department, Biolab Sanus Farmacêutica, Av. Brigadeiro Faria Lima, São Paulo 4509, SP, Brazil; camartins@biolabfarma.com.br (C.P.M.P.); tacastro@biolabfarma.com.br (T.L.d.C.); jbrunetti@biolabfarma.com.br (J.d.V.B.); acunha@biolabfarma.com.br (A.S.C.); ataira@biolabfarma.com.br (A.M.T.); akummer@biolabfarma.com.br (A.M.K.)

**Keywords:** menopause, perimenopause, hyaluronic acid, vaginal dryness, dyspareunia

## Abstract

**Background:** Genitourinary syndrome is a common, chronic condition associated with estrogen deficiency that negatively impacts vaginal health, sexual function, and quality of life in peri- and postmenopausal women. Non-hormonal vaginal moisturizers represent an important therapeutic option for women who cannot or do not wish to use estrogen therapy. **Objective:** The aim of this study was to evaluate the safety and efficacy of Provagin^®^, a hyaluronic acid–based non-hormonal vaginal moisturizer, in postmenopausal women with mild to moderate vaginal dryness. **Methods**: This was a prospective, single-center, open-label, before-and-after clinical study with sexually active postmenopausal women. Participants applied 3 g of Provagin^®^ intravaginally every three days for 22 ± 2 days. Clinical assessments were conducted at baseline, 24 and 72 h after first application, and after 6 and 22 days of use. Outcomes included vaginal pH, vaginal microbiota, epithelial integrity, participant-reported symptoms (vaginal dryness, dyspareunia, and pruritus), and adverse events. **Results:** A total of 45 participants completed the study. Vaginal pH remained stable throughout the study period. No clinically relevant changes were observed in vaginal microbiota, except for a significant increase in *Lactobacillus* spp. counts. Epithelial integrity was preserved at all time points. Significant improvements in participant-reported symptoms were observed as early as 24 h after first use and continued through the study, with mean vaginal dryness scores decreasing by 69.3%. **Conclusions**: Provagin^®^ demonstrated a safety profile and was associated with improvements of genitourinary syndrome of menopause (GSM), without affecting vaginal pH, microbiota composition, or vaginal epithelial integrity. These findings support the use of Provagin^®^ as a safe, non-hormonal therapeutic option for postmenopausal women with GSM.

## 1. Introduction

The genitourinary syndrome of menopause (GSM) is associated with estrogen loss due to the onset of perimenopause (characterized by differences in menstrual cycle ≥ 7 days until the 12-month period since the last menstruation) or menopause or anti-estrogenic therapies [1,2,3]. It is a chronic and progressive disease, including vulvovaginal dryness, dyspareunia, itching, and burning [4]. The constellation of symptoms negatively affects the sexual life impairing orgasm, libido, desire, sexual satisfaction, and intimacy [5]. The menopausal transition typically occurs between 40 and 60 years of age, and approximately 50% of women experience menopausal symptoms, with management strategies depending on symptom severity [6,7]. However, the true burden of GSM is likely underestimated due to insufficient awareness among healthcare providers and the frequent normalization of symptoms as an unavoidable aspect of aging, which contributes to delayed diagnosis and treatment [1].

First-line therapies consist of the use of vulvar and vaginal lubricants and moisturizers [8]. Topical estrogens are considered the treatment gold standard, with various formulations available, but some women have contraindications or some concerns about hormone use [9]. Non-pharmacological treatments, such as vaginal moisturizers, are beneficial, reduce GSM symptoms, and are a good choice for women who cannot or do not want to use estrogen therapy [10]. The treatment aims to relieve the symptoms and mimic the lubrication and integrity of the mucosa in premenopausal period [11].

Hyaluronic acid (HA) is found in the extracellular matrix of the skin and conjunctive tissues, and its use is established as safe and effective as vaginal estrogens [12,13]. Its effect is related to the ability to adhere to the vaginal wall and bind to water, thus enhancing the hydration of the tissue [14]. The aim of this study was to assess the performance and safety of Provagin^®^, a hyaluronic acid (HA)-based vaginal moisturizer, in alleviating the symptoms of genitourinary syndrome of menopause (GSM) in postmenopausal women with mild to moderate vaginal dryness.

## 2. Materials and Methods

### 2.1. Study Design and Participants

This was a prospective, single-center, open-label, before-and-after clinical study. The study protocol was reviewed and approved on 18 October 2024 (no.: 7.168.299) by the Ethics Committee and all participants provided written informed consent. The study adhered to the ethical principles outlined in the Declaration of Helsinki, in accordance with applicable regulatory requirements, including CNS Resolution No. 466/12, and in compliance with ICH E6: Good Clinical Practice and the Document of the Americas. The study was registered in ClinicalTrials (NCT06564883).

This clinical investigation was designed as a performance and safety study to fulfill the regulatory requirements for the maintenance of registration for a Class III medical device. Previously marketed as a cosmetic, the product underwent reclassification under the current Brazilian Health Regulatory Agency (ANVISA) framework, necessitating clinical evidence of its appropriate use in women with GSM. The sample size of 50 participants was determined based on regulatory considerations for established medical devices, rather than a power calculation. This sample size was considered sufficient to provide a robust assessment of safety and clinical performance under real-use conditions.

To minimize the potential impact of participant withdrawals or non-evaluable cases, the study was designed to include 50 participants, allowing for an anticipated dropout rate of approximately 20%. This approach was intended to ensure that at least 40 participants would complete the study and provided sufficient data for the planned safety and performance assessments.

The study population included sexually active (at least once a week) postmenopausal women defined as 12 or more months since last spontaneous menstrual period, aged 50 to 60 years, without systemic or topical hormone replacement therapy (HRT) in the previous six months, presenting mild to moderate vaginal dryness (≥0.5 and <7.5), according to the Visual Analog Scale (VAS).

Exclusion criteria were clinical signs of vaginal disorders, use of topical or systemic medications that could interfere with the study outcomes, including corticosteroids, immunosuppressants, or antihistamines. In addition, participants were excluded if they used of vaginal moisturizers and/or intimate lubricants within five days before the baseline visit; sexual intercourse within 48 h before the baseline visit, a diagnosis of urogenital or vaginal infection within the previous 30 days, and the use of topical or systemic antibiotics, antifungals, hormone-based creams, or treatments for vaginal atrophy within the previous 30 days were also considered exclusion criteria.

### 2.2. Study Product and Formulation

Provagin^®^ (Biolab Sanus Farmacêutica Ltda, São Paulo, SP, Brazil) vaginal gel is a Class III medical device in accordance with the Brazilian Health Regulatory Agency.

The investigational product is a non-hormonal intravaginal moisturizing gel based on hyaluronic acid, formulated with purified water, disodium edetate, glycerol, hydroxyethyl cellulose, benzalkonium chloride, sodium hyaluronate, and lactic acid. It is presented in a 30 g package containing 10 applicators, with a recommendation for intravaginal application every 2–3 days.

### 2.3. Intervention

Participants were instructed to apply the investigational product (3 g) once every three days for a total treatment period of 22 ± 2 days. Each application consisted of intravaginal administration of the product using a vaginal applicator (Biolab Sanus Farmacêutica Ltda, São Paulo, SP, Brazil), with subsequent digital spreading to the inner surface of the labia minora and the vulva. Study product compliance was monitored using a participant diary and product weighing.

To ensure robust safety monitoring, all participants were explicitly instructed to contact the research center immediately upon the occurrence of any sign or symptom, regardless of whether it coincided with a scheduled clinical visit. To facilitate this communication, the investigational product label was designed to include not only the product’s characteristics but also the research center’s contact telephone number, ensuring that participants had immediate access to the clinical team for any eventuality or to report potential adverse events.

To ensure scientific integrity and minimize potential bias, the independent research center was granted full autonomy in the development of the operational protocol, participant recruitment, and the execution of the clinical study, including both clinical and statistical analyses and the drafting of the final clinical report.

The sponsor’s role was primarily focused on quality assurance, providing oversight through Good Clinical Practice (GCP) monitoring and periodic source-data verification to ensure protocol compliance and data accuracy. Additionally, the sponsor reviewed the protocol to ratify that the planned analyses were aligned with the study objectives and, upon completion of the report, performed a comprehensive data review to certify the accuracy of the findings and ensure that the results supported the intended regulatory claims and performance requirements.

### 2.4. Study Assessment

#### 2.4.1. Gynecological Clinical Assessment

Participants underwent a gynecological clinical evaluation performed by a qualified gynecologist at the baseline visit (T_0_), before the use of the investigational product, to confirm eligibility criteria and to conduct the initial safety assessment. Subsequent evaluations were performed at 24 (T_24h_) and 72 (T_72h_) hours after the first application, as well as after 6 (T_6_) and 22 (T_22_) days of product use, to monitor the occurrence of potential adverse events, sensations of discomfort, and to confirm correct use of the investigational product. At each visit, a visual examination of the vulva, vagina, and cervix was conducted using a speculum. Adverse events (AEs) were also recorded throughout the study.

The investigator specifically questioned participants about new symptoms since the previous visit, cross-referencing this information with the participant’s daily diary. All reported adverse events were monitored by the clinical staff until complete resolution. All clinical assessments were performed by the same qualified and trained gynecologist to ensure consistency across visits and reduce inter-examiner variability.

#### 2.4.2. pH Measures

Vaginal pH measurements were performed by a gynecologist using colorimetric indicator strips (range 0 to 14), applied to the lateral vagina wall. Each pH value is associated with a specific color, allowing classification of the sample based on a standardized reference scale. Lower values indicated higher acidity, and higher values were associated with increased alkalinity.

Vaginal pH measurements were conducted at two times: baseline (T_0_), prior to the use of the investigational product, and at the final visit (T_22_), after 22 ± 2 days of use of the investigational product.

#### 2.4.3. Microbiological Analysis

Microbiota samples were collected by a gynecologist using a non-invasive procedure, consisting of a vaginal smear obtained with a sterile swab. The vaginal microbiota analysis included the identification and quantification of *Candida albicans*, *Escherichia coli*, *Gardnerella vaginalis*, *Lactobacillus* spp., and mesophilic microorganisms. Samples were cultured on selective and differential media according to the microorganism of interest.

*Candida albicans* was cultured on CHROMagar Candida and incubated under aerobic conditions at 20–25 °C for 3 days. *Escherichia coli* was cultured on eosin methylene blue (EMB) agar and incubated aerobically at 30–35 °C for 3 days. *Gardnerella vaginalis* was cultured on Casman agar supplemented with 5% sheep blood and incubated at 30–35 °C for 3 days under anaerobic conditions with 5% CO_2_. *Lactobacillus* spp. was cultured on Mann, Rogosa and Sharpe (MRS) agar and incubated anaerobically at 30–35 °C for 3 days. Mesophilic microorganisms were cultured on tryptic soy agar (TSA) and incubated aerobically at 30–35 °C for 3 days.

Vaginal microbiota samples were collected at two times: baseline (T_0_), prior to the use of the investigational product, and at the final visit (T_22_).

#### 2.4.4. Epithelial Integrity Questionnaire

Epithelial integrity was assessed by a single trained gynecologist using standardized epithelial integrity evaluation criteria. The assessment was based on the epithelial integrity domain of the Vaginal Health Index (VHI), which consisted of a five-point ordinal scale with higher scores indicating better epithelial integrity [15]. Scores ranged from 1 to 5 and were defined as follows:

- Score 1: presence of petechiae before contact.
- Score 2: bleeding upon minimal contact.
- Score 3: bleeding upon scraping.
- Score 4: thin but non-friable mucosa.
- Score 5: normal and non-friable mucosa.

The epithelial integrity questionnaire was completed at the following time points: baseline (T_0_), prior to the use of the investigational product; 24 h after the first application (T_24h_); 72 h after the first application (T_72h_); after 6 ± 2 days of product use (T_6_); and at the final visit, after 22 ± 2 days of use of the investigational product (T_22_).

#### 2.4.5. Perceived Efficacy Questionnaire

Subjective outcomes, influenced by patient perception, remain the most widely used tools in the scientific evaluation of GSM. Given that the primary goal of treating GSM is the alleviation of symptoms as perceived by the patient, these subjective assessments are not only appropriate but central to evaluating clinical performance [16].

The perceived efficacy questionnaire was administered at baseline (T_0_), 24 h (T_24h_) and 72 h (T_72h_) after the first application of the investigational product, as well as after 6 days (T_6_) and 22 days (T_22_) of product use. Participants were asked to rate their symptoms of vaginal dryness, pain, and/or discomfort during sexual intercourse, and vaginal pruritus using Visual Analog Scales (VASs).

**Figure 1:**
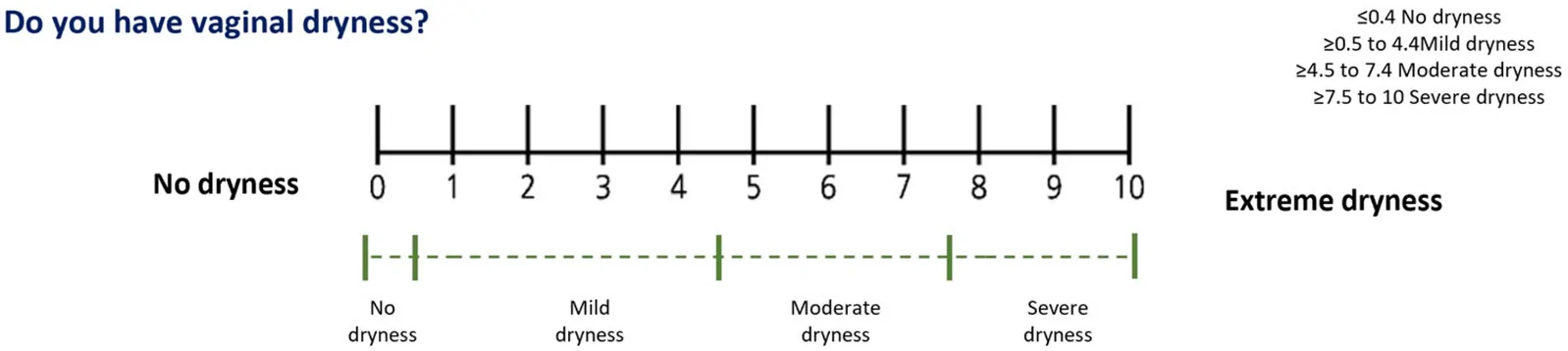
Perceived questionnaire efficacy. Applied to each participant at T_0_, T_24h_, T_72h_, T_6d_, and T_22d_.

Vaginal dryness was classified according to VAS scores as follows: ≤0.4, no dryness; ≥0.5 to 4.4, mild dryness; ≥4.5 to 7.4, moderate dryness; and ≥7.5 to 10, severe dryness, according to Figure 1.

Pain and/or vaginal discomfort during sexual intercourse were assessed on a VAS ranging from 0 (no pain and/or discomfort) to 10 (extreme pain and/or discomfort). Vaginal pruritus was evaluated using a VAS ranging from 0 (no pruritus) to 10 (extreme pruritus).

### 2.5. Statistical Analysis

Data normality was assessed using the Shapiro–Wilk test. For vaginal pH measurements, vaginal microbiological analyses, and epithelial integrity questionnaire scores, comparisons between each post-baseline time point and baseline were performed using the Wilcoxon signed-rank test. For participant-reported efficacy outcomes, one-way ANOVA or the Friedman test was performed, depending on whether the data followed a normal or non-normal distribution, respectively. Pairwise comparison between different time points was performed with a Bonferroni correction for multiple comparisons.

## 3. Results

**Figure 2:**
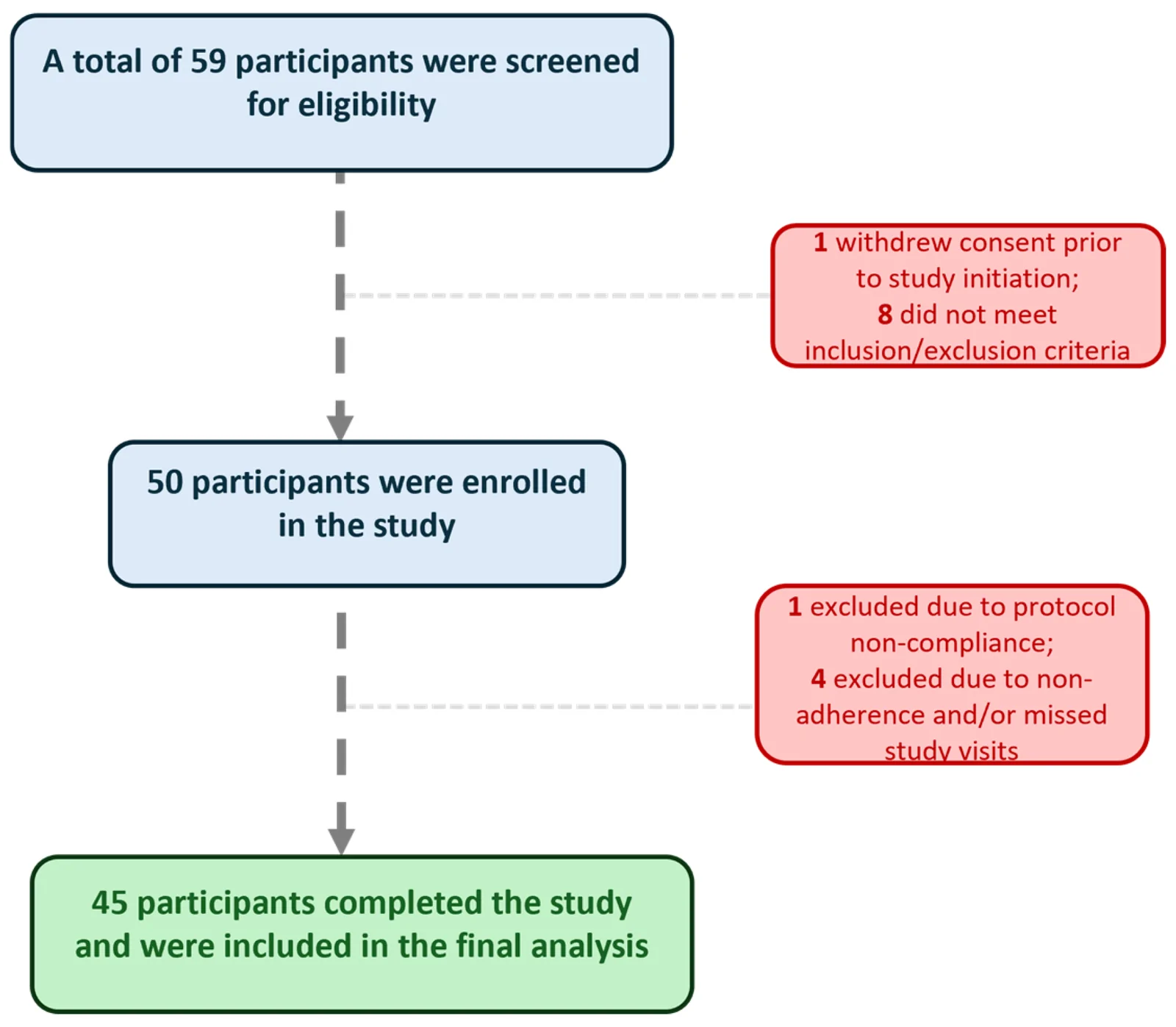
Flowchart of participant screening.

A total of 59 participants were screened for eligibility. Eight did not meet the inclusion and/or exclusion criteria, and one participant withdrew consent prior to study initiation. Consequently, 50 participants were enrolled in the study. During the follow-up period, four participants were excluded due to non-adherence and/or missed study visits, and one participant was excluded because of protocol non-compliance. Therefore, a total of 45 participants completed the study and were included in the final analysis (Figure 2).

### 3.1. pH Measurements

The difference between baseline and T_22_ was not statistically significant (Wilcoxon signed-rank test, *p* = 0.488), indicating that the investigational product did not significantly alter vaginal pH after 22 days of use. The mean pH value was 4.7 at baseline and 4.8 at T_22_, corresponding to a mean change of +0.1 (Table 1).

### 3.2. Microbiological Analysis

**Figure 3:**
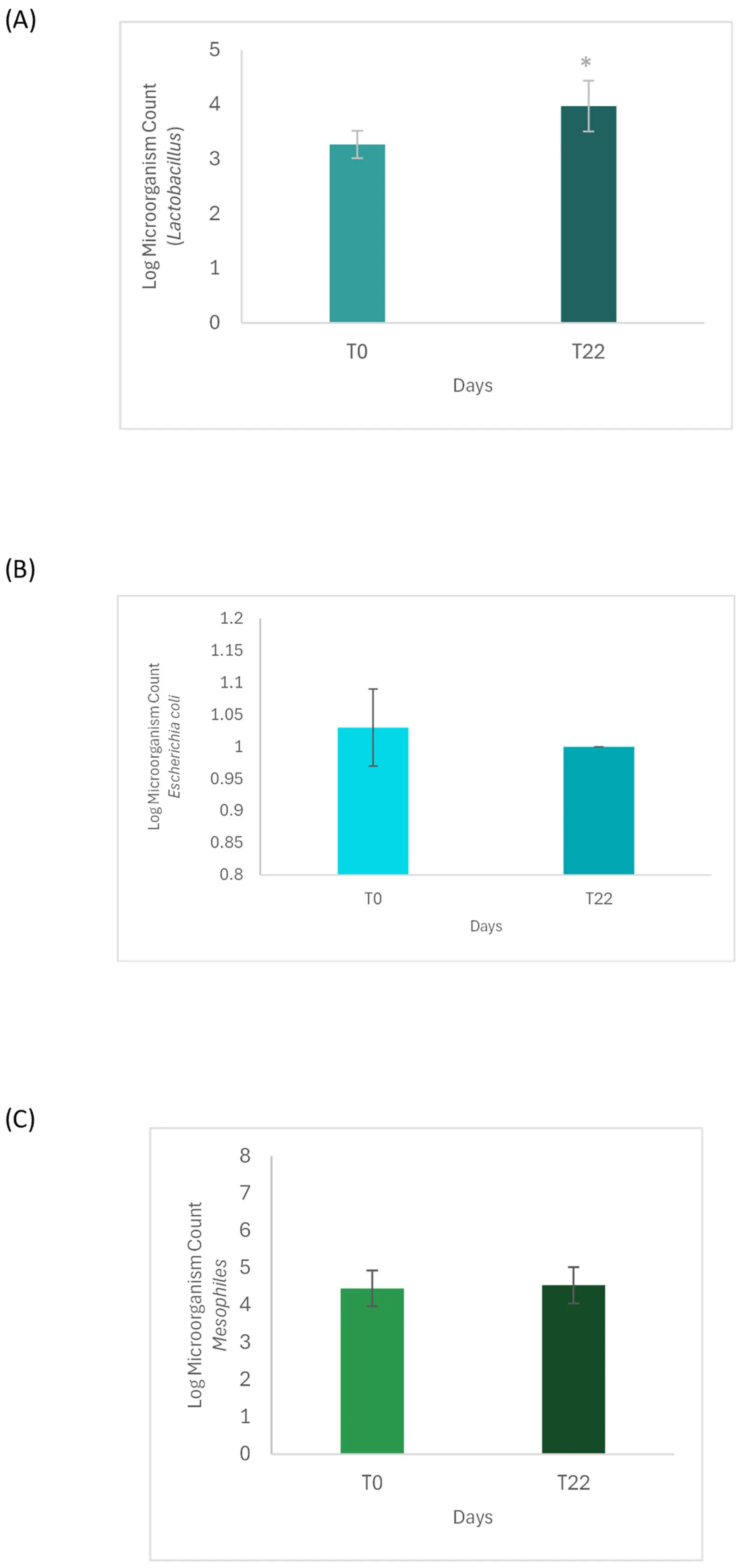
Comparative counts of microorganisms at baseline (T_0_) and after 22 days of product use (T_22_) in the study population (*n* = 45). Data are presented as mean ± standard error (SE). (**A**) *Lactobacillus* spp., (**B**) *Escherichia coli*, and (**C**) mesophilic microorganisms. * *p* = 0.002, *n* = 45.

The study revealed that the proposed non-hormonal vaginal moisturizer did not produce statistically significant changes in the logarithm count of *E. coli* (*p* = 0.317) or mesophilic bacteria (*p* = 0.744). However, significant differences were found in *Lactobacillus* spp., with a mean increase of 21% (*p* = 0.002). *C. albicans* and *Gardnerella vaginalis* were not detected in any participant samples (Figure 3).

### 3.3. Epithelial Integrity

No evidence of vaginal mucosal abnormalities was observed at baseline (T_0_). Also, no abnormalities were detected at 72 h following administration of the investigational product or after 6 days of use, when compared with baseline (T_0_). Furthermore, no statistically significant differences in epithelial integrity were identified at 24 h post-application or after 22 days of product use relative to baseline (T_0_) (Table 2).

### 3.4. Efficacy Perceived

**Figure 4:**
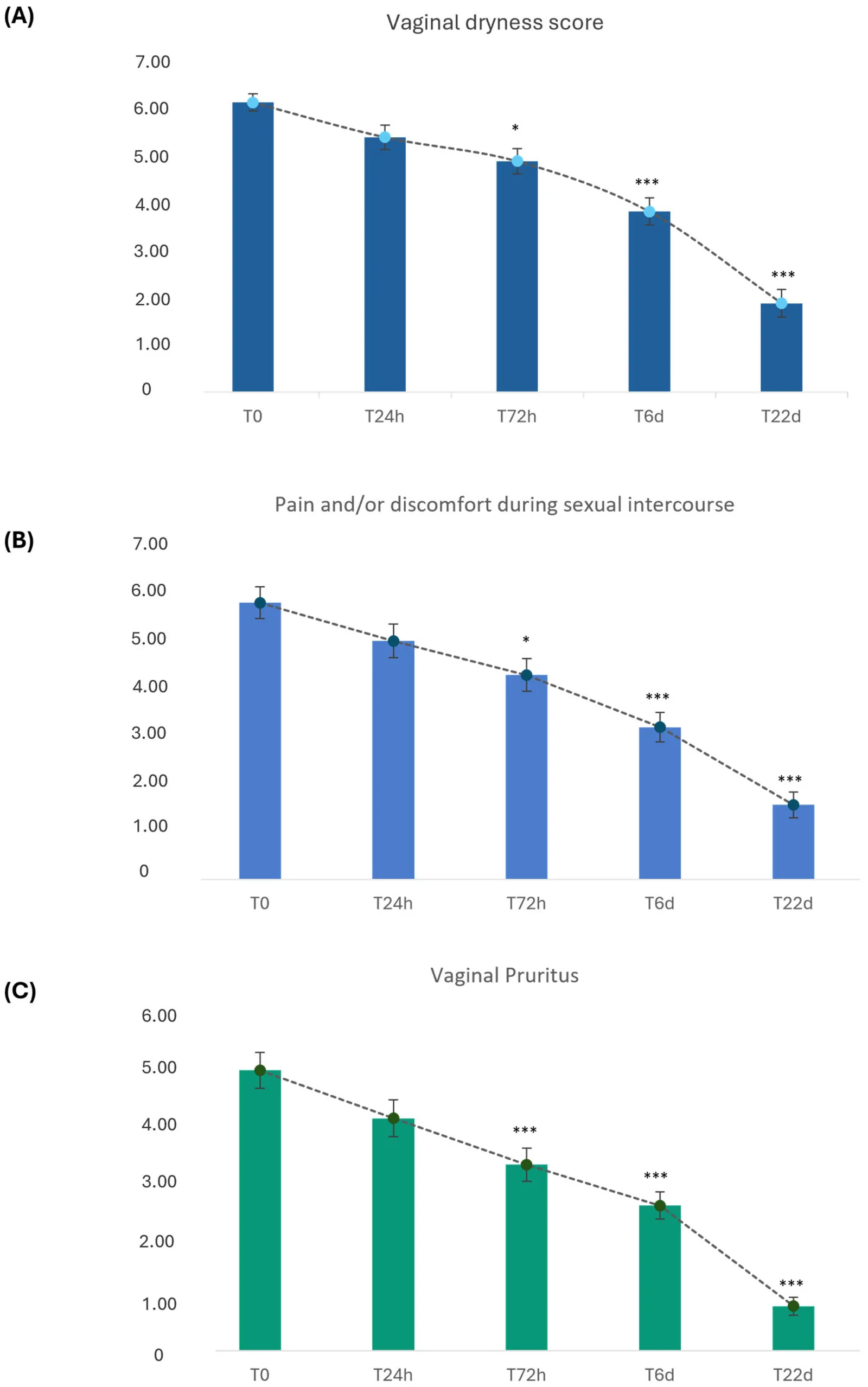
Mean perceived efficacy reported by the 45 participants over time. Error bars represent the standard error (SE). Significant reductions were observed compared with baseline (* *p* < 0.05; *** *p* < 0.001). (**A**) Mean vaginal dryness score over time. (**B**) Pain and/or discomfort during sexual intercourse. (**C**) Vaginal pruritus.

Symptoms associated with GSM, including vaginal dryness, vaginal itching, and dyspareunia, showed statistically significant improvement after 24 and 72 h of investigational product use, as well as after 6 and 22 days, compared with baseline (Figure 4 and Table 3).

#### 3.4.1. Vaginal Dryness

At baseline (T_0_), the mean vaginal dryness score was 6.16 (SE = 0.18). A progressive reduction was observed over time, with mean scores decreasing to 5.42 at 24 h, 4.91 at 72 h, 3.84 at Day 6, and 1.89 at Day 22. This corresponded to mean percentage reductions of 11.9%, 20.2%, 37.5%, and 69.3%, respectively.

The vaginal dryness score differed statistically across the five time points during the use of investigational product (Friedman test; X2 (4) = 139.052; *p* < 0.001), with a significant reduction observed from 72 h and at subsequent time points compared with baseline. Interestingly, the results showed that the vaginal dryness score after 22 days of product application was improved significantly between other time points (between T_22_ and T_6d_: *p* < 0.05; T_22_ and T_72h_: *p* < 0.001; T_22_–T_24h_: *p* < 0.001; T_22_ and T_0_: *p* < 0.001).

#### 3.4.2. Dyspareunia

The study participants reported that at baseline the mean score for dyspareunia was 5.78. Scores progressively decreased to 4.98 at 24 h, 4.27 at 72 h, 3.18 at Day 6, and 1.56 at Day 22. The percentage of participants experiencing symptom reduction was 42.2% at 24 h, 62.2% at 72 h, 91.1% at Day 6, and 93.3% at Day 22. No cases of symptom worsening were reported.

The dyspareunia score differed statistically across the five time points during the use of investigational product (Friedman test; X2 (4) = 135.691; *p* < 0.001), with post hoc analysis indicating a significant reduction from 72 h and at subsequent time points compared with baseline (*p* = 0.002 at 72 h and *p* < 0.001 at both 6 days and 22 days vs. baseline).

#### 3.4.3. Vaginal Pruritus

The mean pruritus score at baseline was 5.02 (SE = 0.32). A consistent decrease was observed over time, with mean scores of 4.16 at 24 h, 3.33 at 72 h, 2.60 at Day 6, and 0.80 at Day 22. The proportion of participants reporting improvement increased from 48.9% at 24 h to 95.6% on Day 22. Only one participant (2.2%) reported a transient worsening of pruritus after the first application, which resolved spontaneously within approximately 40 min, with no recurrence following subsequent applications 24 h.

The pruritus score differed statistically across the five time points during the use of investigational product (Friedman test; X2 (4) = 134.158; *p* < 0.001), with post hoc analysis indicating a significant reduction from 72 h and at subsequent time points compared with baseline (all *p* < 0.001 for the three time point). Consistent with the pattern observed for vaginal dryness, the score at 22 days was significantly lower than at all other time points (*p* = 0.027 between T_22_ and T_6_; *p* < 0.001 for the comparisons between T_22_ and T_72h_, T_24h_ and baseline).

### 3.5. Adverse Events

Six participants (12% of the enrolled population) experienced at least one adverse event. One participant reported an event of moderate intensity (dark urine and uterine pain), while the remaining events were mild (diarrhea, *n* = 2; petechiae, *n* = 2; and pruritus, *n* = 1).

Following medical evaluation and causality assessment, none of the reported adverse events were considered related to the investigational product. The events were classified as: not clearly attributable (*n* = 2), excluded/unrelated (*n* = 2), and unlikely (*n* = 2). No serious adverse events were reported, and no participants discontinued the treatment due to safety concerns.

All adverse events were monitored and managed by the clinical site staff until total resolution of the problem was achieved, ensuring participant safety and the integrity of the safety data.

## 4. Discussion

The present study demonstrated significant improvements in GSM symptoms following 22 days of treatment, with marked reduction in vaginal dryness, dyspareunia and pruritus. These findings are highly relevant because the primary goal of treating GSM is to alleviate symptoms as well as to facilitate comfortable sexual activity, and treatment should be approached stepwise based on symptom severity. Therefore, the clinical significance of the observed relief, particularly the rapid onset within 72 h, aligns with the fundamental objectives of GSM management [8].

The selection of sexually active women aged 50–60 was a deliberate methodological decision intended to maximize the internal validity of this clinical investigation. While we acknowledge that this phenotypically homogeneous cohort does not represent the entire spectrum of women with GSM, it provided the necessary sensitivity to assess the rapid-onset effects of the formulation in a population with high clinical demand for non-hormonal alternatives.

Additionally, no abnormalities in vaginal mucosa were observed, indicating that the products preserve vaginal epithelial integrity, pH levels and microbiota profiles within physiological ranges. Throughout the study, no participant exhibited clinical signs or experienced cutaneous discomfort related to the investigational product, and no clinically significant adverse events were observed.

Estrogen decrease is the main trigger of menopausal symptoms leading to headaches, hot flashes, mood disturbance, sexual impairment, negative impact on quality of life, and genitourinary discomfort [17]. The GSM symptoms are caused by a decrease in estrogen levels [18], This results in reduced elasticity of the vaginal epithelium, regression of the labia minora, and alterations in the vaginal microbiota due to changes in the squamous epithelium that reduce glycogen levels, leading to a decrease in *Lactobacillus* spp. and an increase in vaginal pH [19]. These changes impair quality of life related to sex, enhance the discomfort, facilitate lesions in the mucosa due to the dryness, diminish local defense, and increase the susceptibility to infections [20].

Besides being a part of vaginal microbiota, *Lactobacillus* may regulate pH level due to lactic acid production that catabolizes glycogen, decreasing the vaginal pH level to 3.5–4.5. Therefore, in the context of estrogen deficiency and reduced glycogen availability, preserving an acidic pH becomes even more critical to help maintain microbial balance and protect vaginal health [21]. In our trial, no statistically significant differences were observed in vaginal pH between baseline and Day 22. The maintenance of a physiologically acidic vaginal suggests that the product supports the maintenance of selected beneficial microorganisms (*Lactobacillus*). Preservation of an acidic pH is essential for vaginal health, particularly in postmenopausal women, as it supports the growth and activity of protective *Lactobacillus* species and contributes to the inhibition of pathogenic microorganisms.

*Lactobacilli* are key components of the vaginal commensal flora, with well-established benefits for the female reproductive tract, and disruption of this microbiota has been linked to menopausal vaginal symptoms [22,23]. Microbiological findings further support the product’s safety. No significant changes were observed in *Escherichia coli* or mesophilic bacteria counts, and neither *Candida albicans* nor *Gardnerella vaginalis* was detected in any sample.

The observed increase in *Lactobacillus* spp. suggests that the formulation supports a favorable environment for selected beneficial microorganisms. However, it is important to interpret these findings with caution. Our study utilized conventional culture techniques, which are limited to identifying a specific subset of the vaginal microbiota.

While *Lactobacillus* counts increased, the pH remained stable within a physiologically acidic range (4.7 to 4.8). This suggests that the product, which contains lactic acid, helps maintain the vaginal buffering capacity, preventing the alkalinity typically seen in postmenopausal women without disrupting the ecosystem. This finding is not unexpected, as vaginal pH in postmenopausal women is influenced by multiple factors, including estrogen status, epithelial maturation, glycogen availability, and host-related buffering mechanisms, and therefore may not directly reflect changes in microbiota composition alone [24].

About 50% of postmenopausal women experience symptoms that impair their quality of life and sexual functions [12], but some of them prefer and/or must adopt non-hormonal treatments. Previous studies with vaginal moisturizer containing HA have already demonstrated the effectiveness of acid hyaluronic gel for the treatment of GSM [25,26,27]. In our trial, clinically and statistically significant improvements were observed in GSM-related symptoms, including vaginal dryness, dyspareunia, and pruritus. Symptom relief was evident as soon as the treatment started and continued to improve over the 22 days.

By day 22, vaginal dryness scores decreased by 69.3%. Dyspareunia improved in 93% of participants, while pruritus showed a marked and sustained reduction, with improvement reported by 95.6% of participants. Importantly, no cases of sustained symptom worsening were observed. In addition to the relative reductions of 69.3%, 73.1%, and 84.1% for dryness, dyspareunia, and pruritus, respectively, the absolute reductions exceeded four points on the 10-point VAS for all three symptoms, supporting the clinical relevance of the observed treatment effects beyond statistical significance alone. The early onset and progressive magnitude of symptom relief support the hydrating and bioadhesive properties of hyaluronic acid, which is known for its high water-retention capacity and its role in tissue hydration and elasticity [28].

The clinical significance of these findings is underscored by the magnitude of symptom relief. It is important to note that validated Minimal Clinically Important Differences (MCIDs) for VAS scores in GSM research are currently unavailable in the literature [29,30]. Nevertheless, the observed improvements in this study, specifically the 69.3% reduction in vaginal dryness and the 73.1% reduction in dyspareunia, far exceed the 30% threshold often cited as clinically meaningful in VAS-based symptom assessments [31,32]. The fact that over 93% of participants reported symptomatic improvement reinforces that the statistical significance observed aligns with a substantial clinical benefit for postmenopausal women seeking non-hormonal relief.

The findings of this study support the use of non-hormonal moisturizers as a strategy for GSM management. However, it is important to recognize that symptomatic relief can be further enhanced through complementary non-pharmacological approaches. Recent contemporary evidence suggests that pelvic floor rehabilitation, such as Kegel exercises, plays a significant role in improving GSM-related symptoms and sexual quality of life, whether used alone or in combination with local therapies [33,34].

Taken together, the absence of negative effects on vaginal pH, microbiota, and epithelial integrity, combined with quick significant symptomatic improvement and a favorable safety profile, indicates that Provagin^®^ performs as a non-hormonal vaginal moisturizer associated with symptomatic improvement. These findings are particularly relevant for postmenopausal women who seek alternatives to hormonal therapies or who have contraindications to estrogen use.

The study has some limitations: the study was single-arm and lacked a comparator group, which limits the ability to attribute observed improvements exclusively to the investigational product. Additionally, the sample size of 50 participants was determined by regulatory standards rather than a priori power calculation. The microbiological analysis was limited to conventional culture techniques, providing a partial view of the vaginal ecosystem compared to molecular sequencing. Nevertheless, within the context of a medical device clinical investigation designed to demonstrate safety and performance, the results are robust and clinically meaningful. Importantly, this study was primarily designed as a clinical performance and safety investigation to fulfill the regulatory requirements for a reclassified Class III medical device, rather than a statistically powered efficacy trial. Non-specific effects cannot be excluded due to the uncontrolled study design, and to confirm any efficacy, randomized controlled trials are necessary.

## 5. Conclusions

While the benefits of HA are documented, this study contributes additional evidence regarding the rapid onset of action (relief within 72 h) for this specific formulation. This clinical investigation preliminary demonstrated that the hyaluronic acid-based vaginal moisturizer is safe, well tolerated, and associated with improvements in symptoms associated with genitourinary syndrome of menopause. These observations support the clinical performance of the device under real-use conditions for its intended regulatory purpose. As part of a Clinical Investigation Dossier for Medical Devices, the evidence generated supported the maintenance of Provagin^®^ registration, confirming compliance with applicable regulatory requirements for safety and performance. This clinical investigation provides preliminary evidence that the moisturizer was associated with observed improvements in GSM symptoms. These findings support the clinical performance of the device under real-use conditions for its intended regulatory purpose.

## Figures and Tables

**Table 1:** Mean vaginal pH values in the study participants (*n* = 45) at baseline (T_0_) and after 22 days of product use (T_22_), including the comparison between time points and the corresponding statistical analysis.

Statistic	T_0_	T_22_	T_22_–T_0_
*n*	45	45	Not applicable
Mean pH	4.7	4.8	0.1
Standard error	0.1	0.1	0.1
% mean variation			1.7
% participants with a reduction			24.4
% participants without difference			48.9
% participants with pH increase			26.7
*p*-value			0.488

**Table 2:** Clinical assessment of vaginal mucosal epithelial integrity in the 45 study participants at baseline (T_0_), 24 h (T_24h_), 72 h (T_72h_), 6 days (T_6d_), and 22 days (T_22d_). NC: Not Calculated.

**Epithelial Integrity**	**Time**	**Normal Mucosa % (*n*)**	**Not Friable, Thin Epithelium% (*n*)**	**Bleeds with Scraping, % (*n*)**	**Bleeds with Light Contact % (*n*)**	**Petechiae Noted Before Contact % (*n*)**	***p*-Value**
	T_0_	100% (45)	0% (0)	0% (0)	0% (0)	0% (0)	-
	T_24h_	95.6% (43)	0% (0)	0% (0)	0% (0)	4.4% (2)	0.346
	T_72h_	100% (45)	0% (0)	0% (0)	0% (0)	0% (0)	NC
	T_6d_	100% (45)	0% (0)	0% (0)	0% (0)	0% (0)	NC
	T_22d_	93.3% (42)	6.7% (3)	0% (0)	0% (0)	0% (0)	0.149

**Table 3:** Temporal changes in vaginal symptoms over 22 days among the 45 study participants. Data are expressed as mean ± standard error (SE). Percentage reduction and participant response categories are calculated relative to baseline (T_0_). (**A**) Vaginal dryness, (**B**) pain and/or vaginal discomfort during sexual intercourse over time, and (**C**) vaginal pruritus.

(A) Vaginal Dryness					
Parameter	T_0_	T_24h_	T_72h_	T_6d_	T_22d_
**Mean**	6.16	5.42	4.91	3.84	1.89
**Standard Error**	0.18	0.26	0.27	0.29	0.29
**Mean Reduction (%)**	-	11.9	20.2	37.5	69.3
**Participants with reduction (%)**	-	35.6	60.0	88.9	95.6
**No change (%)**	-	64.4	40.0	11.1	4.4
**Increase (%)**	-	0	0	0	0
***p*** **-value**	-	1.0	0.005	<0.001	<0.001
**(B) Pain and/or Vaginal Discomfort During Sexual Intercourse**					
**Parameter**	**T_0_**	**T_24h_**	**T_72h_**	**T_6d_**	**T_22d_**
**Mean**	5.78	4.98	4.27	3.18	1.56
**Standard Error**	0.33	0.35	0.34	0.31	0.27
**Mean Reduction (%)**	-	13.8	26.2	45.0	73.1
**Participants with reduction (%)**	-	42.2	62.2	91.1	93.3
**No change (%)**	-	57.8	37.8	8.9	6.7
**Increase (%)**	-	0	0	0	0
***p*** **-value**	-	0.255	0.002	<0.001	<0.001
**(C) Vaginal Pruritus**					
**Parameter**	**T_0_**	**T_24h_**	**T_72h_**	**T_6d_**	**T_22d_**
**Mean**	5.02	4.16	3.33	2.60	0.80
**Standard Error**	0.32	0.33	0.30	0.24	0.16
**Mean Reduction (%)**	-	17.3	33.6	48.2	84.1
**Participants with reduction (%)**	-	48.9	66.7	82.2	95.6
**No change (%)**	-	48.9	33.3	17.8	4.4
**Increase (%)**	-	2.2	0	0	0
***p*** **-value**	-	0.278	<0.001	<0.001	<0.001

## Data Availability

The raw data supporting the conclusions of this article will be made available by the authors on request.
